# Oral microbial extracellular DNA initiates periodontitis through gingival degradation by fibroblast-derived cathepsin K in mice

**DOI:** 10.1038/s42003-022-03896-7

**Published:** 2022-09-14

**Authors:** Takeru Kondo, Hiroko Okawa, Akishige Hokugo, Bhumika Shokeen, Oskar Sundberg, Yiying Zheng, Charles E. McKenna, Renate Lux, Ichiro Nishimura

**Affiliations:** 1 Weintraub Center for Reconstructive Biotechnology, Division of Regenerative & Reconstructive Sciences, UCLA School of Dentistry, Los Angeles, CA 90095 USA; 2 Division of Molecular and Regenerative Prosthodontics, Tohoku University Graduate School of Dentistry, Sendai, Miyagi 980-8575 Japan; 3 Regenerative Bioengineering and Repair Laboratory, Division of Plastic and Reconstructive Surgery, Department of Surgery, David Geffen School of Medicine at UCLA, Los Angeles, CA 90095 USA; 4 Section of Biosystems and Function, UCLA School of Dentistry, Los Angeles, CA 90095 USA; 5 Department of Chemistry, Dana and David Dornsife College of Letters, Arts and Sciences, University of Southern California, Los Angeles, CA 90089 USA

**Keywords:** Biofilms, Dental diseases

## Abstract

Periodontitis is a highly prevalent disease leading to uncontrolled osteoclastic jawbone resorption and ultimately edentulism; however, the disease onset mechanism has not been fully elucidated. Here we propose a mechanism for initial pathology based on results obtained using a recently developed Osteoadsorptive Fluogenic Sentinel (OFS) probe that emits a fluorescent signal triggered by cathepsin K (Ctsk) activity. In a ligature-induced mouse model of periodontitis, a strong OFS signal is observed before the establishment of chronic inflammation and bone resorption. Single cell RNA sequencing shows gingival fibroblasts to be the primary cellular source of early *Ctsk*. The in vivo OFS signal is activated when Toll-Like Receptor 9 (TLR9) ligand or oral biofilm extracellular DNA (eDNA) is topically applied to the mouse palatal gingiva. This previously unrecognized interaction between oral microbial eDNA and Ctsk of gingival fibroblasts provides a pathological mechanism for disease initiation and a strategic basis for early diagnosis and treatment of periodontitis.

## Abstract (web summary)

A highly sensitive imaging probe shows that oral microbial extracellular DNA triggers early activity of Ctsk in gingival fibroblasts, initiating periodontitis in mice.

## Introduction

Periodontal disease is a highly prevalent non-communicable inflammatory disease of the tooth-supporting tissues that affects ~70% of adults over 65 years old in the United States^1^, and significantly contributes to the global health burden^2^. The pivotal devastation of periodontitis is uncontrolled tooth-supporting jawbone resorption by overly activated osteoclasts, which is strongly correlated with dental morbidity^3^. It has been postulated that dysbiotic shifts of oral commensal bacterial communities cause aberrant oral barrier immunity^4^, including local differentiation of Th17 cells^5^ and mobilization of neutrophils^6^. These clusters of highly potent immune cells not only develop and sustain chronic inflammation in the oral barrier tissue but also stimulate localized osteoclastogenesis. The clinical diagnosis of periodontitis is currently based, in large part, on dental radiographs of the alveolar bone morphological changes^7^. However, when bone resorption is clearly detected radiographically, the disease has already progressed and irreversible changes to the bone level, which often lead to tooth loss, have manifested.

The degree of periodontal pocket formation generated by the loss of gingival attachment to the affected dentition also serves as a critical diagnostic measure for the severity of periodontitis^8^. The periodontal pocket provides an anaerobic environment suitable for colonization by periodontal pathogens that can trigger dysbiotic shifts of oral commensal microbial communities. The periodontal pocket is formed by the disarrangement and degradation of gingival and periodontal ligament extracellular matrix (ECM) and the apical extension of the junctional epithelium^9^. Initial gingival inflammatory responses are prompted by the presence of oral biofilm communities at the tooth-tissue interface of the gingival sulcus^10^. A study of human cadaver tissues revealed that periodontal pocket formation was accompanied by degradative changes in gingival and periodontal ligament fibroblasts and collagenous ECM^11^. However, a knowledge gap still exists: how gingival and periodontal ligament ECM is degraded prior to the development of chronic oral barrier inflammation; and how oral microorganisms contribute to the initiation of periodontal pocket development.

Osteoclasts secrete cathepsin K (Ctsk), a cysteine protease that degrades the bone collagen matrix^12^. Ctsk was also found to be secreted by other cell types that contributed to pathological processes of tendon^13^ and vascular tissues^14^. We have recently developed a bone-targeting, Ctsk-activated fluorescent sensor, Osteoadsorptive Fluorogenic Sentinel (OFS) probes, in which a bisphosphonate (BP) moiety is covalently attached to a fluorophore linked via a Ctsk octapeptide substrate to an internal quencher that suppresses external fluorescence by Förster resonance energy transfer (FRET). The inclusion of the BP modifier results in adsorption of OFS to bone surfaces, where an external fluorescent signal is activated if locally released Ctsk cleaves the linker^15^. The sensitivity of OFS fluorescence activation has been demonstrated in a humanized mouse model of multiple myeloma where the OFS signal was detected at the orthotropic grafted site of luciferase-tagged multiple myeloma cells prior to the detection of luciferase activity^15^.

In this investigation, we employed OFS probes in an experimentally induced murine periodontitis model to elucidate the pathological mechanism of periodontitis initiation by detecting early Ctsk activity prior to the manifestation of radiographic bone changes. This in vivo research platform surprisingly identified early Ctsk activity of gingival fibroblasts immediately after the ligature placement. Furthermore, the in vivo OFS signal was detected when microbial extracellular DNA (eDNA) was topically applied to mouse palatal gingiva. This previously unrecognized relationship between eDNA and gingival Ctsk provides evidence for the processes involved in the initial progression of periodontitis leading to effective diagnostic and therapeutic modalities.

## Results

### Ctsk activation prior to inflammation and bone resorption in a ligature-induced mouse periodontitis model

**Fig. 1:**
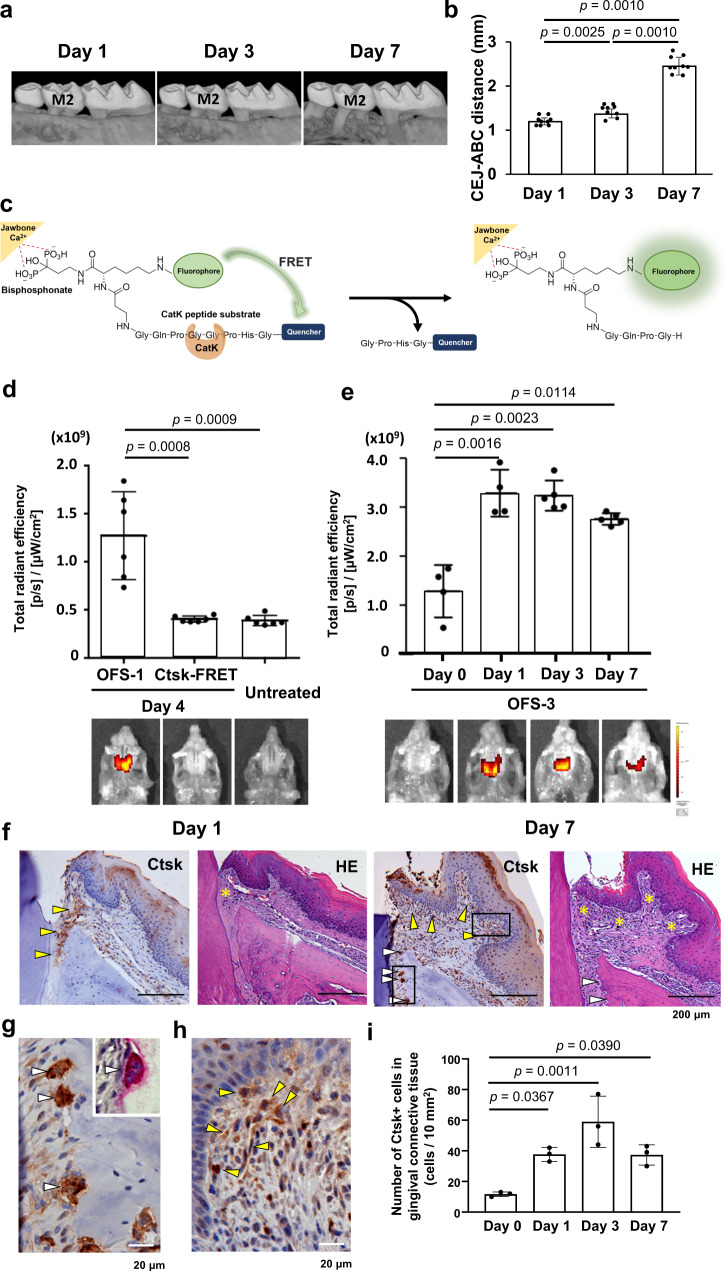
Early cathepsin K (Ctsk) activation in the ligature-induced periodontitis in mice. **a** The ligature (5.0 silk suture) was placed around the maxillary second molar (M2). Representative microCT images of the maxilla taken from the lateral view at the 1, 3, and 7 days after ligature placement. **b** Alveolar bone loss was measured at the middle of the second molar from cementoenamel junction (CEJ) to the alveolar bone crest (ABC) (*n* = 9). **c** The Osteoadsorptive Fluorogenic Sentinel (OFS) probe. OFS binds strongly to bone, where it remains quenched until activation by Ctsk through cleavage of a linker region containing the Ctsk substrate motif. **d** Day 4 of ligature placement, OFS-1 signal was detected but the fluorescent signal was not detected from a commercially available Ctsk FRET probe without a bisphosphonate bone anchoring element (SensoLyte 520, Anaspec, Fremont, CA) (*n* = 6). **e** Representative images showing Ctsk activation of OFS-3 fluorescence detected by an IVIS Spectrum system at 0, 1, 3, and 7 days after ligature placement. Quantification of the fluorescent signal was performed by region-of-interest placement to capture the entire palate, and results were represented as the total fluorescent signal (*n* = 4–5). **f** Immunohistochemical staining for Ctsk, counterstaining with methylene blue, and HE staining of the periodontal tissue at the 1 and 7 days after ligature placement. *Yellow arrows* indicate the Ctsk positive cells in the gingival and periodontal ligament connective tissue. *White arrows* indicate the Ctsk-positive osteoclasts on the alveolar bone and osteoclastic lacunae. *Yellow asterisks* indicate disintegrated gingival connective tissue. **g** A high-magnification photomicrograph of Day 7 IHC depicting Ctsk+ osteoclasts (while arrows). The insert shows TRAP+ osteoclasts. **h** A high-magnification photomicrograph of Day 7 IHC depicting Ctsk+ connective tissue cells (*yellow arrows*). **i** The number of Ctsk+ cells in gingival connective tissue (*n* = 3). ANOVA with Tukey’s multiple-comparison test (**b**, **e**, **i**) and Student’s *t* test (**d**). Data are presented as mean values ± SD; *p* < 0.05 was considered significant. The source data are provided in Supplementary Data 1, **b**, **d**, **e**, **i**.

We used the ligature-induced mouse periodontitis model (Supplementary Fig. 1a), which was previously reported to establish chronic inflammation and alveolar bone resorption 5–7 days after silk suture placement around the maxillary second molar^5,16^. Gingival swelling was observed 3 days after the ligature placement (Supplementary Fig. 1b) and localized to the ligature placement side (Supplementary Fig. 1c). The induction of inflammatory cytokine gene expression was also progressively increased in the gingiva of the ligature placement side over the experimental period (Supplementary Fig. 1d). The MicroCT image evaluation showed clear radiographic alveolar bone reduction on day 7 (Fig. 1a, b).

To monitor disease initiation and progression, we determined Ctsk activity using the OFS probe (Fig. 1c)^15^. We synthesized a fluorescein-OFS probe (OFS-1) with a 5-FAM fluorophore which emits at 518 nm, quenched by BHQ-1, and a far-red OFS probe (OFS-3) which emits at 650 nm (SulfoCy5 quenched by BBQ-650) (Supplementary Fig. 2a–c). Both OFS-1 and OFS-3 gave rise to similar results. Unlike a commercially available Ctsk FRET probe (SensoLyte 520, Anaspec, Fremont, CA) without bone retention properties, the incorporation of a bone anchoring property into the OFS probe allowed localization to the jawbone surface, which provided sensitive detection of local Ctsk activity (Fig. 1d).

OFS probe administration by intravenous (IV) injection was combined with the ligature-induced periodontitis mouse model. This in vivo research platform clearly demonstrated that Ctsk was activated from Day 1 after ligature placement (Fig. 1e). These results suggest that Ctsk activation occurred in the very early stages of periodontitis development prior to the establishment of chronic gingival inflammation and alveolar bone resorption.

Immunohistochemical (IHC) staining revealed the presence of Ctsk in the periodontal ligament and gingival connective tissue on Day 1 (Fig. 1f). On Day 7, Ctsk was strongly observed in osteoclasts on the surface of alveolar bone (Fig. 1f, g). It was noted that the Ctsk+ cells in the gingival connective tissue (Fig. 1h) remained in the area beneath the epithelial layer from Day 1 to Day 7 (Fig. 1i). These results indicated that Ctsk was secreted by cells in the gingiva connective tissue other than osteoclasts.

### Ctsk induced periodontal connective tissue degradation

**Fig. 2:**
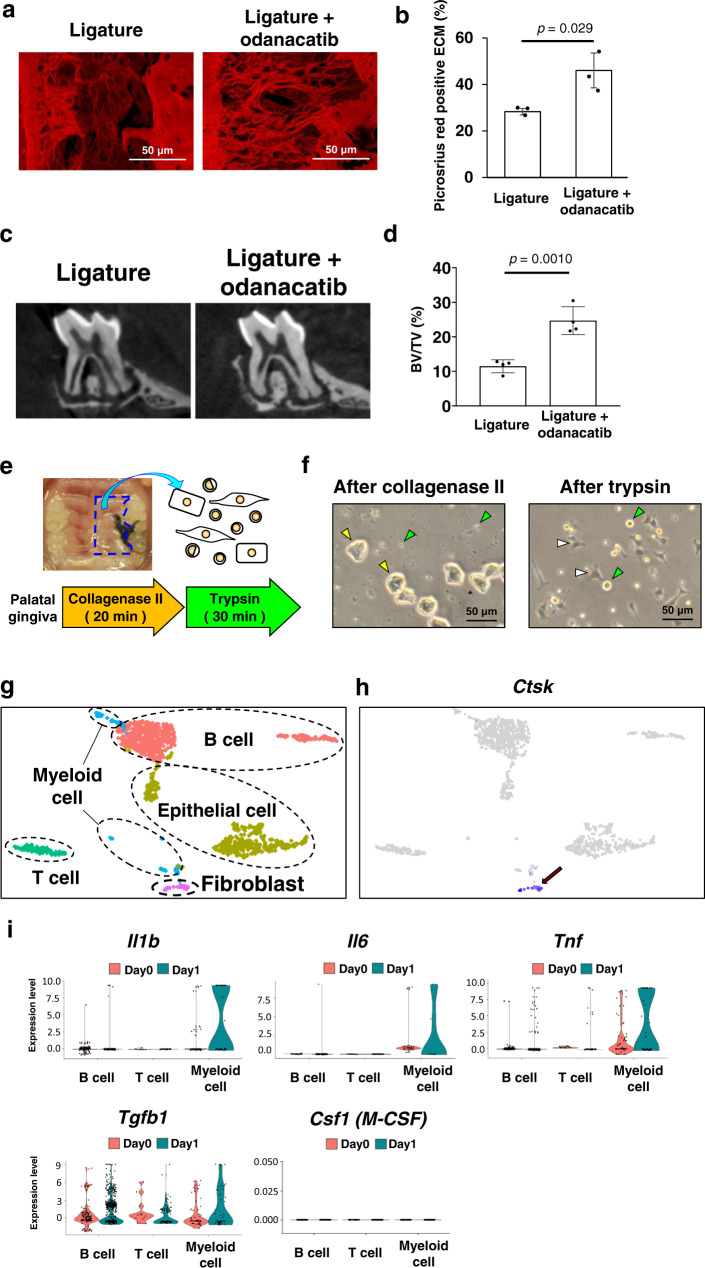
The pathological role of Ctsk and its cellular source in mouse gingiva. **a** Representative fluorescent picrosirius red images of the gingival connective tissue and periodontal ligament at 7 days after ligature placement with or without administration of the Ctsk inhibitor odanacatib (scale bars: 50 μm). **b** The picrosirius-stained collagen structure in the periodontal ligament connective tissue area was measured as percentage of the area between tooth surface and the surface of alveolar bone (*n* = 3). **c** Representative microCT cross-sectional images of second molar 7 days after ligature placement with or without odanacatib administration. **d** The average bone volume/total volume (BV/TV) in the alveolar bone on the buccal and palatal side of the second molar as measured from the apex of the root to the cementoenamel junction (*n* = 4). **e** Single-cell dissociation method for the ligature side of the palate at 1 day after ligature placement. **f** Phase-contrast images of dissociated cells after collagenase II treatment and trypsin treatment (scale bars: 50 μm). *Yelllow arrows* indicate epithelial cells. *Green arrows* indicate immune cells. *White arrows* indicate fibroblasts. **g** Single-cell RNA-sequencing (scRNA-seq) *t*-SNE projection plots showing major classes of dissociated cells at 1 day after ligature placement. Colors indicate cell type (*Red*: B cell, *yellow*: epithelial cell, *green*: T cell, *blue*: myeloid cell, *pink*: fibroblast). **h** scRNA-seq *t*-SNE projection plots showing transcript accumulation for *Ctsk* genes predominantly in fibroblasts (arrow). **i** Comparative evaluation of scRNA-seq of gingival cells derived from untreated (Day 0) and 1 day after the ligature placement (Day 1). The early reaction of the ligature placement was depicted by upregulation of inflammatory cytokines *Il1b*, *Il6* and *Tnf* by myeloid cells. The expression pattern of *Tgfb1* and *Csf1 (M-CSF)* was not affected by ligature placement for 1 day. Student’s *t* test (**b**, **d**). Data are presented as mean values ± SD; *p* < 0.05 was considered significant. The source data are provided in Supplementary Data 1, **b**, **d**.

To explore the pathological function of Ctsk in periodontitis, odanacatib, an inhibitor of Ctsk^17^, was administered to mice upon ligature placement. Picrosirius red staining showed that ligature placement induced severe degradation of gingival connective tissue and periodontal ligament on Day 7, whereas it was significantly attenuated by the oral gavage administration of odanacatib (Fig. 2a, b). Moreover, bone resorption was also suppressed by odanacatib (Fig. 2c, d) as expected^18^. These results suggest that Ctsk could play important roles in periodontal tissue ECM degradation as well as osteoclastic alveolar bone resorption.

### Identification of the cellular source of Ctsk in the early periodontitis lesion

To identify the cellular source of Ctsk in the gingival tissue at Day 1 of ligature placement, we performed single-cell RNA-sequencing (scRNA-seq). The ligature side of the palatal gingiva at 1 day after ligature placement was harvested and exposed to two types of enzymes (Collagenase II and Trypsin) (Fig. 2e, f). We identified five major cell types dissociated from the palatal gingiva by cluster mapping using the expression of lineage-specific genes: *Cd19* for B cells; *Cd3e* for T cells; *Lyz2* for myeloid cells; *Krt5* for epithelial cells; and *Col1a1* for fibroblasts (Fig. 2g and Supplementary Fig. 3). scRNA-seq data were screened for *Ctsk* and a high level of *Ctsk* expression was observed in the fibroblast cluster. Our data indicated that gingival fibroblasts were the major cellular source of Ctsk in the initial stage of periodontitis development (Fig. 2h). We also obtained gingival cells from naïve control mice designated as Day 0. scRNA-seq of gingival immune cells indicated the increased pro-inflammatory cytokines: *Il1b, Il6,* and *Tnf* in the myeloid cells from Day 0 to Day 1 (Fig. 2i), consistent with early myeloid cell infiltration^19^.

### TLR9 stimulation induced Ctsk activation and gingival ECM degradation

**Fig. 3:**
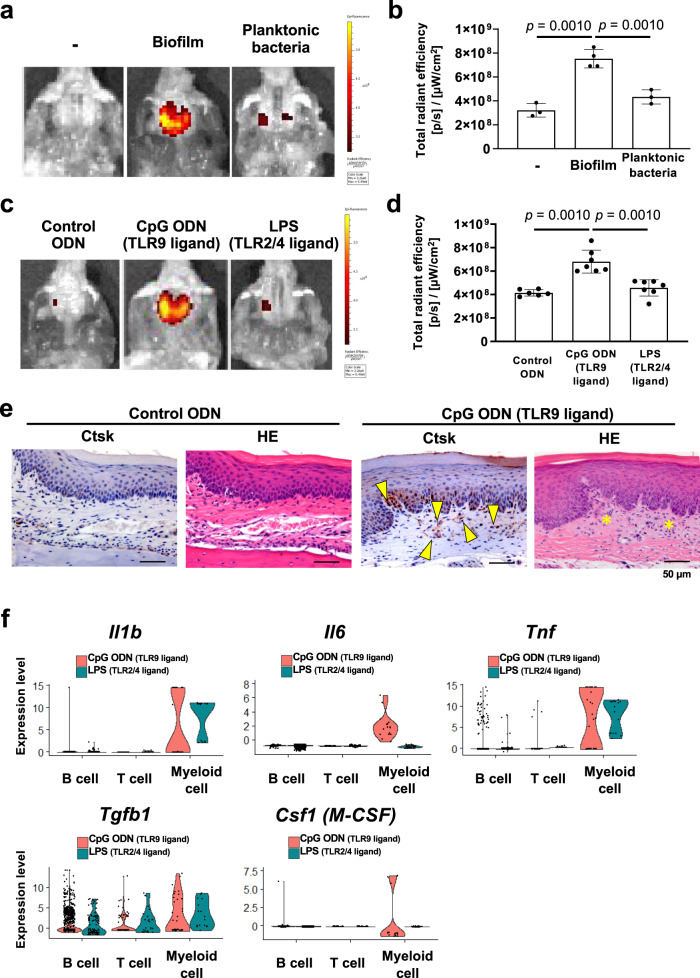
Topical application of human oral biofilm and biofilm components to activate Ctsk in palatal tissue of mice. **a** Cultured human oral microbial biofilm or planktonic bacteria was topically applied to the mouse palatal gingiva for 1 h covered by an oral appliance. OFS-1 was IV injected 3 days after the topical application of human microbial samples and 24 h later, the Ctsk activation was detected by OFS-1 fluorescence. **b** Quantification of the OFS-1 fluorescent signal at 4 days after topical application of cultured oral microbial biofilms or planktonic bacteria to the palate (*n* = 3–4). **c** Representative images showing OFS-1-derived fluorescent signal 4 days after the topical application of control ODN, CpG ODN (TLR9 ligand), or LPS (TLR2/4 ligand) solution to the palatal gingiva (*n* = 6–7). **d** Quantification of the OFS-1 fluorescent signal after topical application of control ODN, CpG ODN, or LPS to the mouse palatal gingiva. **e** Immunohistochemical staining for Ctsk and HE staining of the periodontal tissue at 4 days after topical application of control ODN or CpG ODN (scale bars: 50 μm). *Yellow arrows* indicate Ctsk positive cells in the connective tissue. *Yellow asterisks* indicate the disintegrated gingival connective tissue. **f** scRNA-seq violin plots showing gene expression levels of *Il1b*, *Il6*, *Tnf*, and *Csf1(M-CSF)* in myeloid cells which were collected from the palatal gingival tissue 4 days after topical application of CpG ODN or LPS solution, which did not affect *Tgfb1* expression. ANOVA with Tukey’s multiple-comparison test (**b**, **d**). Figure presented as mean values ± SD; *p* < 0.05 was considered significant. The source data are provided in Supplementary Data 1, **b**, **d**.

To investigate the role of oral microbial biofilms in the activation of gingival Ctsk, in the present study cultured human oral microbial biofilm containing a mixture of live bacteria and extracellular polymeric substances (EPS)^20^ or planktonic bacteria were topically applied to the mouse palatal gingiva covered by a custom-made oral appliance for 1 h. Three days after the topical application of human oral microbial biofilm or planktonic bacteria, OFS was administered by IV injection. The resulting OFS fluorescence was measured the following day, which revealed that human oral microbial biofilm activated the OFS signal, while OFS activation was minimal in the corresponding planktonic bacteria topical application group (Fig. 3a, b). From these results, we hypothesized that components of the EPS matrix induced the gingival Ctsk activation.

Toll-like receptors (TLRs) are known to play critical roles in the defense against microorganisms and to activate inflammatory responses^21^. This study topically applied synthetic CpG DNA oligonucleotide (CpG ODN) stimulating TLR9^22^ and *Porphyromonas gingivalis* lipopolysaccharide (LPS) activating TLR2/4^23^ to the mouse palate for 1 h, followed by administration of OFS probe on Day 3 and examination of OFS activation on Day 4. The TLR9 ligand CpG ODN induced the OFS signal significantly more than the TLR2/4 ligand LPS (Fig. 3c, d). IHC analysis showed that CpG ODN activated Ctsk in gingival fibroblasts localized below the epithelial layer (Fig. 3e). The area of gingival ECM with Ctsk+ fibroblasts appeared to have lost typical collagen architecture, suggesting localized gingival ECM degradation (Fig. 3e).

The cells dissociated from the palatal gingiva that were exposed to CpG ODN or LPS were analyzed by scRNA-seq (Fig. 3f). CpG ODN-treatment induced higher expression of *Il1b, Il6, Tnf,* and *Csf1* (*M-CSF)* in myeloid cells compared to LPS, while similar *Tgfb1* expression between the CpG ODN and LPS groups was observed in B cells, T cells and myeloid cells (Fig. 3f). In addition, we compared the scRNA-seq data of LPS, CpG ODN to Day 0 and Day 1 of ligature placement. While Day 0 scRNA seq did not show noticeable cytokine gene expression, we found that the gene expression pattern of Day 1 scRNA seq (Fig. 2i) is similar to that of CpG ODN scRNA seq (Fig. 3f). Thus, we propose that the similarity in cytokine expression pattern of Day 1 and CpG ODN might indicate that Day 1 inflammation may, at least in part, be induced by microbial DNA-related inflammatory reaction.

### Mechanism of gingival fibroblast Ctsk activation by CpG ODN

TLR9 is predominantly located intracellularly in immune cells including dendritic cells in the oral barrier tissue^24^. Therefore, we initially expected gingival epithelial dendritic cells (Langerhans cells) or oral barrier dendritic cells to be the primary sensor of CpG ODN. Analysis of scRNA-seq data suggested the presence of *Cd207 (Langerin)*+ cells in the myeloid cell cluster of Day 0 and Day 1 gingiva (Supplementary Fig. 4a, d, respectively). The myeloid cell cluster was further subclustered and one subcluster was found to express *Cd207 (Langerin), Epcam,* and *Cd11c* (Supplementary Fig. 4b, e) indicating that Langerhans cells dissociated from gingival epithelium were included in the myeloid cell cluster. The scRNA-seq data of the myeloid cell subclusters were further examined for TLR gene expression (Supplementary Fig. 4c, f) but *Tlr4 or Tlr9* were not detected in the Langerhans cells.

**Fig. 4:**
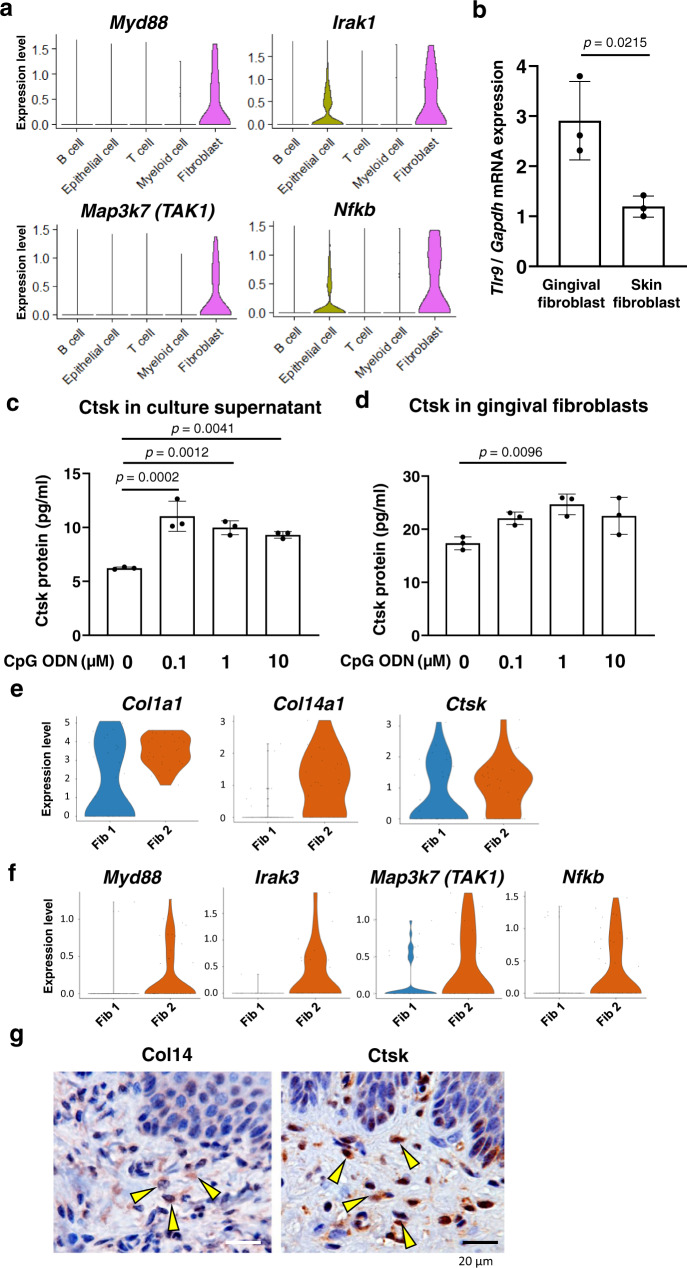
TLR9 in gingival fibroblasts. **a** The expression of TLR-related downstream signal transduction genes in gingival cells harvested 1 day after the ligature placement. scRNA-seq violin plots showing gene expression level of *Myd88*, *Irak1*, *Map3k7 (TAK1),* and *Nfkb* predominantly in fibroblasts. **b** Gene expression of *Tlr9* in gingival fibroblasts and skin fibroblasts was determined by quantitative real-time RT-PCR analysis (*n* = 3). **c** Ctsk in supernatant of mouse gingival fibroblast culture with CpG ODN supplementation determined by ELISA (*n* = 3). **d** Ctsk in the cultured gingival fibroblast with CpG ODN supplementation determined by ELISA (*n* = 3). **e** scRNA-seq of Day 1 fibroblasts revealed two distinct subclusters: Fib 1 and Fib 2. *Col1a1* (type 1 collagen) was expressed by both Fib 1 and Fib 2, while *Col14a1* (type XIV collagen) was exclusively expressed by Fib 2. Fib 1 and Fib 2 expressed *Ctsk* albeit that its expression level was high in Fib 2. **f** Expression of *Myd88*, *Irak1*, *Map3k7 (TAK1),* and *Nfkb* was found exclusively in Fib 2. **g** IHC of type XIV collagen (Col14) and Ctsk showed positive staining in fibroblasts (*yellow arrows*) localized near the gingival epithelial cells. Student’s *t* test (**b**) and ANOVA with Bonferroni’s correction of pairs with the control group of CpG ODN 0 µM (**c**, **d**). Data are presented as mean values ± SD; *p* < 0.05 was considered significant. The source data are provided in Supplementary Data 1, **b**–**d**.

As activation and stability of TLRs are not only regulated by putative gene transcription but also by post-translational modifications such as proteolytic cleavage and phosphorylation^25^, we investigated the gene expression of downstream signal transduction cascades initiated by activated TLR9. We found that the downstream genes *Myd88*, *Irak1*, *Map3k7*(*TAK1),* and *Nfkb* were highly expressed in the fibroblast cluster in our scRNA-seq analysis (Fig. 4a). We thus hypothesized that gingival fibroblasts might possess the TLR9 sensing mechanism. RT-qPCR of primary gingival fibroblasts harvested from untreated control mice showed a high steady-state level of *Tlr9* mRNA (Fig. 4b). To validate the TLR9 function in relation to Ctsk, gingival fibroblasts were cultured in the presence of serially diluted CpG ODN. ELISA of the culture supernatant and the fibroblast homogenate revealed elevated Ctsk levels (Fig. 4c, d, respectively). Ctsk induction within gingival fibroblasts showed a dose-dependent increase with the peak at 1 µM CpG ODN supplementation. By contrast, the Ctsk secretion to the culture supernatant indicated a more sensitive response peaking at 0.1 µM CpG ODN supplementation. The data also indicated that possibly different mechanisms were involved in TLR9-activated secretion and accumulation of Ctsk in gingival fibroblasts.

Fibroblasts express heterogeneous cellular phenotypes and localize as a cluster in the connective tissue. We collected palatal gingival tissue from the alveolar bone surface and thus it is unlikely that the dissociated cells contained periodontal ligament fibroblasts. Nonetheless, we analyzed the Day 1 scRNA-seq data in more detail. To our surprise, we found two distinct clusters of gingival fibroblasts: fibroblast cluster 2 (Fib 2) distinctly expressed type XIV collagen (*Col14a1*) (Fig. 4e). Type XIV collagen is a member of the fibril associated-collagen with interrupted triple helices (FACIT) and has been reported in the upper layer of the dermis close to the epidermis in skin^26^, tendon^27^, and periodontal ligament^28^. The analysis of scRNA-seq suggested that both Fib 1 and Fib 2 expressed *Ctsk* transcripts albeit with a higher expression level in Fib 2 than Fib 1 (Fig. 4e). However, the down steam signal transduction molecules of TLRs were exclusively expressed by Fib 2 (Fig. 4f). IHC using anti-Type XIV collagen (Col14) antibody and anti-Ctsk antibody recognized fibroblasts localization in the gingival connective tissue zone near the epithelial layer of free gingiva and connective tissue papillae (Fig. 4g). This observation suggests that Fib 2 may be the primary source of Ctsk protein synthesis and secretion responding to CpG ODN topically applied to the gingival epithelium.

### Oral microbial eDNA may play a role in the initiation of periodontitis

**Fig. 5:**
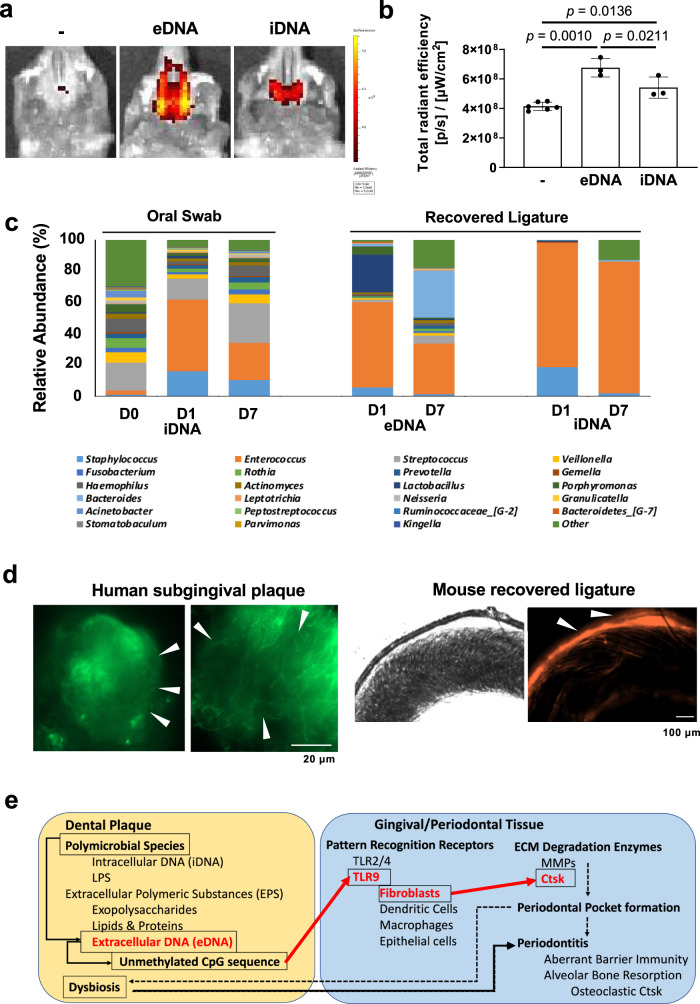
Microbial extracellular DNA (eDNA) is a potent activator of gingival Ctsk. **a** OFS-1 was IV injected 3 days after the 1 h topical application of eDNA or iDNA to the mouse palatal gingiva and the fluorescent signal was recorded 4 days after the eDNA or iDNA topical application. **b** Quantification of OFS-1 fluorescent signal (*n* = 3–6). **c** Relative abundance of microbial genera in the mouse periodontitis model. The palatal gingival swabs were collected before (D0), 1 day (D1), and 7 days (D7) after ligature placement (*n* = 3 per time point). The ligatures were recovered from the mouse maxillary second molar D1 and D7 (*n* = 4 per time point). eDNA and iDNA samples were prepared (*n* = 4 per sample that were combined) and subjected to 16 S rRNA sequencing. **d** SYTOX green-fluorescent images of subgingival plaque collected from human subjects with clinically diagnosed periodontitis (scale bar: 20 μm) and a pair of incident-light and SYTOX orange-fluorescent images of a recovered ligature from the mouse periodontitis model. *Arrowheads* indicate eDNA. **e** The proposed mechanism of periodontitis initiation (red letters and arrows). The present study indicated that eDNA component of the dental plaque biofilm might be readily recognized by gingival fibroblasts through TLR9. Upon the activation, gingival fibroblasts may release Ctsk, resulting in gingival degradation leading to the periodontal pocket formation (dotted line). ANOVA with Tukey’s multiple-comparison test (**b**). Data are presented as mean values ± SD; *p* < 0.05 was considered significant. The source data are provided in Supplementary Data 1, Fig. 5b.

We demonstrated that oral microbial biofilm and unmethylated CpG ODN activated the OFS signal in vivo at significantly higher levels compared to planktonic bacteria (Fig. 3a, b) or LPS (Fig. 3c, d). Microbial DNA is less methylated at its CpG sequences than mammalian genes and thus triggers TLR9 more effectively. To investigate if human oral microbial DNA was a ligand for TLR9, we separately isolated microbial extracellular DNA (eDNA) and microbial intracellular chromosomal DNA (iDNA) from oral microbial biofilms. Microbial eDNA is a pivotal structural component of microbial biofilms^29^ that can be in contact with the gingival tissue. Topical application of both eDNA and iDNA to the mouse palatal gingiva indeed activated Ctsk (Fig. 5a); however, the effect of eDNA was stronger compared to the same amount of iDNA (Fig. 5b).

We then investigated if eDNA was produced in the ligature-induced mouse periodontitis model. Palatal gingival swabs were obtained before (Day 0), Day 1, and Day 7 after ligature placement. The mouse oral microbial samples harvested from the swabs was evaluated by 16 S rRNA sequencing. The oral swab microbial composition at the genus level appeared to be modulated after ligature placement, in which *Enterococcus* was notably increased (Fig. 5c) consistent with a previously published report^5^. However, eDNA was not isolated from the gingival swab samples. By contrast, the ligatures recovered on Day 1 and Day 7 were successfully used to harvest eDNA and iDNA. Oral microbial composition analysis from 16 S rRNA sequencing revealed that the biofilm community associated with the ligature was different from the untreated mouse palatal gingival swab samples (D0); however, showed some resemblance to the palatal gingival swab samples after the ligature placement. The ligature-associated microbial community composition (iDNA) appeared to be less diverse than the corresponding eDNA (Fig. 5c).

SYTOX Green-staining disclosed an eDNA meshwork spreading throughout the EPS of subgingival plaque samples collected from human subjects with diagnosed periodontitis (Fig. 5d). The recovered ligatures from the mouse periodontitis model similarly contained SYTOX Orange-stained eDNA-like structure (Fig. 5d). These data validated the presence of eDNA in human oral biofilms and mouse ligatures recovered from the periodontitis model.

## Discussion

The present study used a recently developed bone-targeting OFS FRET-based detection system of Ctsk activity in vivo and directly implicates fibroblastic Ctsk in the gingival tissue degradation potentially leading to periodontitis. Ctsk is a lysosomal cysteine protease with the strong collagenolytic activity known to mediate bone resorption by osteoclasts^30^. Ctsk in the gingival crevicular fluid of periodontitis patients was elevated compared to healthy patients, which was thought to reflect increased osteoclastic activity in periodontal tissues^31,32^. A role of Ctsk in periodontal bone resorption has been demonstrated using Ctsk knockout mice^33^ and Ctsk inhibitor^18^, suggesting that osteoclast-derived Ctsk plays the predominant role in periodontitis development. The unique observation of this study was the detection of Ctsk activity by OFS and IHC in the gingival connective tissue prior to the establishment of chronic inflammation and osteoclast induction (Fig. 1) and the early cellular source of Ctsk was strongly suggested to be gingival fibroblasts by scRNA-seq analysis (Fig. 2).

Fibroblastic Ctsk has been linked to abnormal connective tissue degradation of synovial tissue of rheumatoid arthritis^34^ and a rare lung nodule formation^35^. In the dermis, Ctsk secretion from fibroblasts only occurs under pathological circumstances such as a scarring^36^ or inflammation^37^. Therefore, we postulated that Ctsk activation by gingival fibroblasts was also activated by pathological stimuli. Periodontitis is caused by oral biofilms that are composed of heterogeneous, polymicrobial communities encased in a matrix of EPS^38^. Using the OFS in vivo research platform, we dissected a microbial component that led to the induction of Ctsk activation and microbial DNA emerged as a potential trigger (Fig. 3). Undermethylated microbial DNA is a ligand for TLR9. In fact, *Tlr9* knockout mice have been shown to be resistant to developing experimental periodontitis upon *P. gingivalis* oral innoculation^39^ or ligature placement^40^, supporting the pathological role of microbial DNA and TLR9 in periodontitis. Intriguingly, stimulation with the TLR2/4 ligand LPS from *P. gingivalis* resulted in a lower early immune response compared to CpG ODN. A reason for this could be that we employed a one-time topical application of LPS compared to previous studies that injected LPS into the gingival tissue 2-3 times a week^41,42^. Nevertheless, our study suggests that TLR9 plays an important role in Ctsk-mediated initiation of periodontitis.

The function of TLR9 has been extensively investigated in primary antigen-presenting cells such as dendritic cells, macrophages, and B cells^43^. However, gingival immune cells including Langerhans cells did not clearly show the expression of *Tlr9* by the scRNA-seq evaluation (Fig. S4). In our study, mouse gingival fibroblasts were shown to express *Tlr9* and the corresponding downstream signal transduction genes (Fig. 4). Furthermore, the treatment with CpG ODN for gingival fibroblasts induced increased protein synthesis and secretion of Ctsk in vitro (Fig. 4). Cultured human gingival and periodontal ligament fibroblasts from periodontitis subjects express TLR2/4^44,45^, however, the expression of TLR9 has not been detected^46^. Recent studies suggested that fibroblasts are heterogeneous in nature^47,48^. Further scRNA-seq evaluation identified two subclusters of gingival fibroblasts: Fib 1 and Fib 2 (Fig. 4e), of which Fib 2 uniquely expressed type XIV collagen and might play a distinct role in microbial DNA-interaction through TLR9 (Fig. 4f). The IHC using anti-type XIV collagen antibody revealed that Fib 2 localize in the gingival connective tissue zone near the epithelial layer of free gingiva and connective tissue papillae, where Ctsk IHC similarly showed positive fibroblasts (Fig. 4g). Taken together, we postulate that a subcluster of gingival fibroblasts (Fib 2) may be directly implicated in the initial gingival connective tissue degradation through the secretion of microbial DNA-induced Ctsk.

The taxonomic composition of the microbial communities colonizing humans and mice was found to differ significantly^49^. While this is not surprising, our 16 S rRNA sequencing analysis demonstrated that the mice used in our experiments harbored many of the same genera that are present in the human oral cavity. Interestingly though, more than half of the DNA in both iDNA and eDNA that was recovered from the ligature corresponded to *Enterococcus* and *Staphylococcus*, two genera that were not prominent members of the oral mouse cavity prior to ligature placement (Fig. 5). These results validate the previously reported mouse oral microbial composition^5^ and suggests that ligature-induced periodontitis in mice may not mirror the human oral microbial communities.

eDNA is a structural component of biofilms that stabilizes and shapes the EPS scaffold^50,51^. In addition, eDNA has a role in bacterial adhesion during the early stages of biofilm formation and maintenance^52,53^, and is thus localized in close contact to the gingival tissue. eDNA is generated by different mechanisms such as lysis of microbes within the biofilm or active release from living cells^54^. Therefore, it is conceivable that the ligature-associated eDNA might represent a mixture of lysed biofilm cells as well as free microbial eDNA from the saliva or other oral sources that could have been trapped in the biofilm accumulated in the ligature.

Although eDNA can clearly be seen as strands between the microbes in human subgingival plaque and recovered ligature from the mouse periodontitis model (Fig. 5), the function of eDNA in the initiation of periodontitis has not yet been investigated. The present study demonstrated that topical application of human eDNA to the mouse palatal gingiva activated gingival Ctsk. Periodontal microbial DNA has been reported to trigger the production of inflammatory mediators in various cell types such as macrophages^55–57^ and gingival fibroblasts^57,58^ through TLR9. The relevance of microbial composition and species responsible for the initiation of periodontitis in humans and mice have been debated as both species harbor distinct microbiomes in their oral cavities. However, if the excreted or secreted microbial eDNA containing consensus unmethylated CpG sequences are involved in periodontal connective tissue degeneration by Ctsk derived from gingival fibroblasts, the presence of specific microbial species may not be required for the initial pathogenesis of periodontitis (Fig. 5e).

In conclusion, we report evidence that early disease pathogenesis of periodontitis involves activation of Ctsk secretion from gingival fibroblasts, which is triggered by oral microbial eDNA in dental plaque. We propose that responses of gingival fibroblasts to microbial eDNA leading to secretion of Ctsk into the connective tissue space are directly implicated in initiating periodontal disease by connective tissue degradation and periodontal pocket formation (Fig. 5e). Our data suggest a basis for early disease diagnosis systems and suggests previously unidentified therapeutic targets for prevention or treatment of this highly prevalent oral disorder.

## Materials and methods

### Animal care

All protocols for animal experiments were reviewed and approved by the University of California Los Angeles (UCLA) Animal Research Committee (ARC# 2003-009) and followed the Public Health Service Policy for the Humane Care and Use of Laboratory Animals and the UCLA Animal Care and Use Training Manual guidelines. C57BL/6 J wild type mice (Jackson Laboratory, Bar Harbor, ME) were used in this study. Animals had free access to regular rodent diet and water ad libitum and were maintained in standard housing conditions with 12-hour-light/dark cycles in the Division of Laboratory Animal Medicine at UCLA. All animal experiments were designed, conducted, and reported following the Animal Research: Reporting of In vivo Experiments guideline (ARRIVE 2.0)^59^.

### Human subjects

All protocols involving human subjects were reviewed and approved by the UCLA Institutional Review Board (UCLA-IRB 11-002483). The participants provided verbal informed consent to take part in the study. Saliva samples were collected from 20- to 40-year-old healthy human subjects. Subgingival plaque samples were collected from 20- to 40-year-old human subjects with clinical diagnosis of periodontitis. The detailed protocols for further sample processing are described below.

### Osteoadsorptive fluorogenic substrate probes

OFS-1 and OFS-3 were synthesized and characterized as described previously^10^. The probes were dissolved in 0.7 mL of 0.9% sodium chloride (NaCl) in water to a concentration of 50 μM and stored at 4 °C in the dark until use.

### Evaluation of gingival swelling, alveolar bone resorption and Ctsk activation in a ligature-induced mouse model of periodontitis

A silk thread was gently tied around the left maxillary second molar of 8- to 12-week-old female C57BL6/J wild type mice under general inhalation anesthesia with isoflurane (Henry Schein, Melville, NY). To characterize Ctsk activation, OFS-1 or OFS-3 was prepared and characterized as previously described^15^ with minor modifications to further improve yield, and 100 µl of 10 µM OFS prepared in 0.9% NaCl solution was injected through the retro-orbital venous plexus one day prior to euthanasia. At 1, 3, 5, and 7 days after the ligature placement, mice were euthanized by 100% CO_2_ inhalation. The maxillary gingival tissues were then digitally photographed and harvested, and the fluorescent signal was measured with the IVIS Spectrum Imaging System (IVIS Lumina II: Perkin Elmer, Waltham, MA). The gingival swelling area was measured using a Java-based image processing program (ImageJ: NIH, Bethesda, MD) and normalized to the circumferential area of the maxillary second molar. After evaluation of Ctsk using the IVIS, the maxillary bones were harvested from each mouse and fixed in 10% buffered formalin (Thermo Fisher Scientific, Waltham, MA). The fixed maxillary bones were X-rayed at an energy level of 60 kV and 166 µA, and 3D images were reconstructed (Skyscan 1275: Bruker, Billerica, MA). Alveolar bone loss was measured at the middle of the second molar from the cementoenamel junction to the alveolar bone crest. Statistical analysis was performed using two-way analysis of variance with Tukey’s multiple-comparison test to assess the difference among multiple experimental groups. *P* < 0.05 was considered as statistically significant.

### Histological and IHC evaluation of ligature-induced periodontitis in mice

The harvested maxillae with gingival tissue at 1, 3, and 7 days after ligature placement were fixed in 10% buffered formalin (Thermo Fisher Scientific, Waltham, MA) and decalcified in 10% EDTA (Sigma-Aldrich, Saint Louis, MO) for 3 weeks. After the decalcification, samples were embedded in paraffin. Histological cross-sections were stained by hematoxylin and eosin (H&E) and evaluated on a light microscope. Adjacent paraffin sections (4 µm) were immunohistochemically stained for Ctsk (anti-cathepsin K antibody #ab19027, Abcam, Waltham, MS) at 1:1000 dilution after heat-induced epitope retrieval procedure followed by the secondary antibody application and diaminobenzidine staining and methylene blue counterstaining. Ctsk+ gingival connective tissue cells were counted in the 10 mm^2^ area.

For some histological sections, IHC analysis of type XIV collagen (COL14A1 Polyclonal antibody, #PA5-49916, Thermo Fisher Scientific, Waltham, MA) at 1:100 dilution was also performed following the protocol. Osteoclasts were evaluated by tartrate-resistant acid phosphatase (TRAP) staining using a commercially available kit (Acid Phosphatase TRAP kit, Sigma-Aldrich, St. Louis, MO) following the manufacturer’s protocol.

### Evaluation of gene expression of *Il1b*, *Il6*, *Il17a,* and *Tnfsf11 (RANKL)* in a ligature-induced mouse model of periodontitis

At 1, 3, and 7 days after ligature placement, total RNA was extracted from the harvested ligature side or non-ligature side of the maxillary gingival tissues with the RNeasy Mini Kit (QIAGEN, Germantown, MD) and quantified with a Thermo Scientific NanoDrop 1000 ultraviolet-visible spectrophotometer (NanoDrop Technologies, Wilmington, DE). After treatment with DNase I (Thermo Fisher Scientific), cDNA was synthesized from 1 µg of total RNA using Super Script III reverse transcriptase (Super Script VILO: Thermo Fisher Scientific).

Taqman-based qRT-PCR was performed using commercially available primer/probe mixes as follows, *Il1b* (Mm00434228_m1, Thermo Fisher Scientific), *Il6* (Mm00446190_m1, Thermo Fisher Scientific), *Il17a* (Mm00439618_m1, Thermo Fisher Scientific) and *Tnfsf11 (RANKL)* (Mm00441908_m1, Thermo Fisher Scientific) in combination with a mouse *Gapdh* internal control mix (Mm99999915_g1, Thermo Fisher Scientific). Target gene expression was quantitatively analyzed using the *ΔΔ*CT method. Statistical analysis was performed using Student’s *t* test to assess the difference between the ligature side group and the non-ligature side group at each time point. *P* < 0.05 was considered as statistically significant.

### Examination of the function of Ctsk in the initial stage of periodontitis

Following ligature placement, mineral oil (Sigma-Aldrich) alone or supplemented with odanacatib (Selleckchem, Houston, TX) at a dose of 90 µg/100 µl was orally administered. Human studies reported a long elimination half-live of odanacatib, which was estimated to be 3 to 4 days^60,61^. In this study, odanacatib was administered once before the ligature placement. The gingival swelling area was measured, and the maxillary bones were scanned by microCT at 7 days after ligature placement as described above. Alveolar bone resorption was measured at the middle of the second molar from the cementoenamel junction to the alveolar bone crest. The average of bone area/total area in the alveolar bone on the buccal and palatal side of the second molar was measured from the apex of the root to the cementoenamel junction.

Harvested maxillae were decalcified and prepared for histological sections. The paraffin sections were stained with picrosirius red (PolyScience, Niles, IL). The collagen fiber structure of the gingival connective tissue and periodontal ligament was evaluated using confocal laser scanning microscopy (SP8: Leica Microsystems, Wetzlar, Germany). Picrosirius red was visualized via excitation with a 20 mW DPSS 561 nm and emission collection at 635-685 nm bandwidth. The connective tissue area was measured (ImageJ) and normalized to the area between tooth surface and the surface of the alveolar bone. Statistical analysis was performed using Student’s *t* test to assess the difference between the experimental groups. *P* < 0.05 was considered as statistically significant.

### Evaluation of steady state gene expression profiles in a ligature-induced mouse model of periodontitis by scRNA-seq

The naïve untreated maxillary gingival tissue (designated as Day 0) and the gingival tissues after 1 day of ligature placement (designated as Day 1) were harvested and subjected to gingival cell dissociation as following.

#### Collagenase II treatment

The tissues were cut into ~1 mm^2^ pieces and placed immediately into 20 ml digestion buffer containing 1 mg/ml collagenase II (Life Technologies, Grand Island, NY), 10 units/ml DNase I (Sigma-Aldrich) and 1% bovine serum albumin (BSA; Sigma-Aldrich) in Dulbecco’s modified Eagle’s medium (DMEM; Life Technologies). The chopped tissues were incubated in the digestion buffer for 20 minutes at 37 °C on a 150 rpm shaker. The tissues were then passed through a 70 µm cell strainer, pelleted at 1500 rpm for 10 minutes at 4 °C before being resuspended in phosphate-buffered saline (PBS; Life Technologies) that was supplemented with 0.04% BSA (Cell suspension A) and counted to generate “Cell suspension A”.

#### Trypsin treatment

The parts of the tissues that did not pass through the 70 µm cell strainer after collagenase II treatment, were subjected to additional incubation in 10 ml of 0.25% trypsin (Life Technologies) and 10 units/ml DNase I for 30 minutes at 37 °C on a 150 rpm shaker. Trypsin was neutralized with 10 ml of fetal bovine serum (FBS; Life Technologies), and the tissues were passed through a 70 µm cell strainer, which was washed with 10 ml DMEM. The collected cells were then pelleted at 1500 rpm for 10 minutes at 4 °C, resuspended in PBS that was supplemented with 0.04% BSA and counted to generate “Cell suspension B”.

Cell suspension A and Cell suspension B were combined into one tube and cell viability was determined. For scRNA-seq, gingival cells were treated with the Chromium single-cell 3’ solution (10X Genomics, San Francisco, CA) using microfluidic partitioning to capture single cells and prepared the barcoded, next-generation sequencing cDNA library (10X Genomics, San Francisco, CA). The Cell Ranger software was used to align reads, generate feature-barcode matrix and perform clustering. The Cell Ranger output of scRNA-seq data was then analyzed using an R toolkit for single-cell genomics (Seurat, https://satijalab.org/seurat/). Cells with fewer than 800 detected genes were discarded. The signature gene was used to identify: B cells (*Cd19*), T cells (*Cd3e*), Myeloid cells (*Lyz2*), epithelial cells (*Krt5*) and fibroblasts (*Col1a1*). The expression of *Ctsk* was determined in each cell cluster. The myeloid cluster and fibroblast cluster was further subclustered for designated analyses.

### Evaluation of Ctsk activation by topical application of cultured oral biofilm or planktonic bacteria

Saliva samples from 20- to 40-year-old healthy human subjects were collected and diluted to 25% with PBS. The diluted saliva was centrifuged at 2600 × *g* for 10 minutes to pellet large debris and eukaryotic cells. Prior to seeding of the oral biofilm, 100 µl of the diluted saliva was grown in 1 ml of SHI medium^62^ for 17–18 hours under anaerobic conditions (10% CO_2_, 10% H_2_, and 80% N_2_). This overnight grown oral microbial community was pelleted and washed with PBS. For biofilm seeding, cells were diluted cells to an optical density at 600 nm of 0.1 into 100% SHI medium supplemented with 5 mM CaCl_2_. Further, 1 ml of this diluted oral community was seeded onto oral appliances which were custom-made of clear dental resin (GC America, Alsip, IL) and incubated under anaerobic conditions at 37 °C for 5 days. Cultured oral community was treated with 10 U/ml DNase I and resuspended in PBS to prepare a planktonic bacteria solution free of EPSs including eDNA.

The palates were covered by an oral appliance with cultured oral biofilm, or three µl of planktonic bacteria solution (3 × 10^7^ CFU) were topically applied to the palate and the palates were covered by an oral appliance. After 1 hour of covering the palate, the oral appliances were removed. One hundred µl of 10 µM OFS solution was injected through the retro-orbital venous plexus one day prior to euthanasia. At 4 days after the topical application of cultured oral biofilm or planktonic bacteria, the OFS fluorescent signal was measured. Statistical analysis was performed using two-way analysis of variance with Tukey’s multiple-comparison test to assess the difference among multiple experimental groups. *P* < 0.05 was considered as statistically significant.

### Evaluation of Ctsk activation by topical application of CpG DNA or LPS

Three µl of 1 µg/ml of CpG ODN (InvivoGen, San Diego, CA), 1 µg/ml of LPS from *P. gingivalis* (InvivoGen) or 1 µg/ml of control ODN (InvivoGen) were topically applied to the palate as described above, and 100 µl of 10 µM OFS solution was injected through the retro-orbital venous plexus one day prior to euthanasia. At 4 days after the topical application of CpG ODN, *P. gingivalis* LPS or control ODN, the OFS fluorescent signal was measured. Statistical analysis was performed using two-way analysis of variance with Tukey’s multiple-comparison test to assess the difference among multiple experimental groups. *P* < 0.05 was considered as statistically significant. In addition, the harvested maxillas were subjected to immunohistochemial staining for Ctsk and stained with HE as described above.

### Evaluation of steady state gene expression profiles in mouse gingival tissues with topical application of CpG DNA or LPS by scRNA-seq

At 4 days after the palatal topical application of CpG ODN or *P. gingivalis* LPS, the maxillary gingival tissues were harvested from freshly isolated mouse maxillas. Single cells were dissociated from the maxillary gingival tissues, and scRNA-seq was performed as described above.

### Evaluation of gene expression of *TLR9* in gingival fibroblasts and skin fibroblasts

Primary gingival fibroblasts or skin fibroblasts from 8- to 12-week-old female wild type mice were cultured using an explant method as previously reported^63^. The cells were cultured in DMEM with 10% FBS and 100 U penicillin/0.1 mg/ml streptomycin (Life Technologies) at 37 °C, 5% CO_2_ in a humidified incubator.

Total RNA was extracted from the gingival fibroblasts or skin fibroblasts, and cDNA was synthesized as described above. Taqman-based RT-qPCR was performed using a commercially available primer/probe mix for *Tlr9* (Mm00446193_m1, Thermo Fisher Scientific). Statistical analysis was performed using Student’s *t* test to assess the difference between the experimental groups. *P* < 0.05 was considered as statistically significant.

### Evaluation of induction of Ctsk secretion or production of Ctsk protein in gingival fibroblasts by CpG DNA

Primary mouse gingival fibroblasts were cultured in DMEM supplemented with 10% FBS and 100 U penicillin/0.1 mg/ml streptomycin in the presence of 0, 0.1, 1 or 10 µg/ml of CpG ODN at 37 °C, 5% CO_2_ in a humidified incubator for 24 hours.

Culture supernatant or cell solution lysed with RIPA Lysis and Extraction buffer (VWR, Radnor, PA, USA) supplemented with a protease and phosphatase inhibitor cocktail (Thermo Fisher Scientific). The Ctsk protein concentration in the culture supernatant or lysed cell solution was determined by a colorimetric method (OD at 450 nm) using a Ctsk ELISA kit (MyBioSource, San Diego, CA, USA). Statistical analysis was performed using one-way analysis of variance with Bonferroni’s multiple-comparison test to assess the difference only of pairs relative to the control group. *P* < 0.05 was considered as statistically significant.

### Extraction of iDNA or eDNA from a human saliva-derived oral microbial community

eDNA and iDNA were extracted from human saliva-derived biofilms. After biofilm growth in 100% SHI medium supplemented with 5 mM CaCl_2_ under anaerobic conditions at 37 °C for 5 days, planktonic cells were removed by gently aspirating the medium and carefully washing once with 500 µl PBS. Following the wash, 250 µl of PBS was added in each well, and microbial cells were harvested by scraping and pipetting with a sterile pipette tip. The bacterial cells were then transferred to an Eppendorf tube and pelleted at 3250 × *g* for 15 minutes at 4 °C for DNA isolation. The supernatant was filtered through 0.22 µm syringe filters to exclude bacterial cells. While the bacterial pellet was used for iDNA isolation, the cell- free supernatant was further processed for eDNA isolation.

#### iDNA extraction

The bacterial pellet was used for iDNA extraction using the Epicentre MasterPure DNA extraction and Purification Kit (Lucigen, Middleton, WI) according to the manufacturer’s instructions^64^.

#### eDNA extraction

eDNA was extracted from the cell-free supernatants of the microbial biofilm according to a previously described protocol^65^. Briefly, the cell-free supernatants (containing eDNA) were mixed with 2 volumes of absolute ethanol and a 1/10th volume of sodium acetate (3 M, pH 5.2) containing 1 mM EDTA. After overnight precipitation at −80 °C, eDNA was pelleted by centrifugation at 13,000 rpm at 4 °C for 20 minutes followed by a wash with ice-cold 70% ethanol. The eDNA was then air dried, dissolved in sterile deionized water and quantified using NanoDrop.

### Evaluation of Ctsk activation by topical application of eDNA or iDNA

Three µl of 1 µg/ml solution of eDNA or iDNA were topically applied to the palate as described above, and 100 µl of 10 µM OFS solution was injected through the retro-orbital venous plexus one day prior to euthanasia. At 4 days after the topical application of eDNA or iDNA, the OFS fluorescent signal was measured. Statistical analysis was performed using two-way analysis of variance with Tukey’s multiple-comparison test to assess the difference among multiple experimental groups. *P* < 0.05 was considered as statistically significant.

### Evaluation of eDNA and iDNA of mouse oral biofilm harvested from recovered ligatures

The ligatures were recovered from the mouse model of periodontitis 1 day (*n* = 4) and 7 days (*n* = 4) after placement. iDNA and eDNA samples were prepared separately as above and subjected to 16 S rRNA sequencing of the V4 region (Laragen, Inc, Culver City, CA). Demultiplexed sequences were imported into Qiime 2 (v2020.11). Low quality sequences containing bases with Phred quality values <20 were trimmed and denoised using the DADA2 package^66^. The amplicon sequence variants generated after the denoising were taxonomically assigned by comparison to the HOMD database.

### SYTOX Green/Orange staining of the subgingival plaque from a periodontitis patient and the recovered ligature

Subgingival plaque was collected from a periodontitis patient and stained with SYTOX Green (Thermo Fisher Scientific). The eDNA scaffold in the plaque was evaluated via fluorescence microscopy (Zeiss Axio Imager M2 with Zen 2.5 pro software). Sytox green was visualized using epifluorescence through a ×100/1.4 Plan Achromat objective at 450–490 nm bandwidth excitation and 500–550 nm bandwidth emission filters. The recovered ligature from the mouse periodontitis model was stained with SYTOX Orange and was visualized similarly but with 538–562 nm bandwidth excitation and 570–640 nm bandwidth emission filter settings.

### Statistics and reproducibility

For statistical analysis, Student’s *t* test or one-way analysis of variance with Tukey’s multiple-comparison test or with Bonferroni correction was performed. A significant difference was defined by *p* < 0.05. The sample size and number of replicates ware reported in the legends of figures. The data were showed as mean and standard deviation.

### Reporting summary

Further information on research design is available in the Nature Research Reporting Summary linked to this article.

## Supplementary information

Supplementary Information

Description of Additional Supplementary Data

Supplementary Data 1

Supplementary Data 2

Supplementary Data 3

Supplementary Data 4

Supplementary Data 5

Supplementary Data S1

Reporting Summary

## Data Availability

The source data supporting the findings of this article are provided in Supplementary Data 1–5 and S1. Single-cell RNA sequence data are available at GEO: GSE201108 and GSE201109. All other data are available from the corresponding author upon reasonable request.
