# Supplementary material for: Oral microbial extracellular DNA initiates periodontitis through gingival degradation by fibroblast-derived cathepsin K in mice
Source: Commun Biol. 2022 Sep 14;5:962. doi: 10.1038/s42003-022-03896-7 (PMC9474870; doi:10.1038/s42003-022-03896-7)
Supplement: Supplementary file 1 — Supplementary Information [file 42003_2022_3896_MOESM1_ESM.pdf]

## Supplementary Information

# Oral Microbial Extracellular DNA Initiates Periodontitis through Gingival Degradation by Fibroblast-derived Cathepsin K in Mice

Takeru Kondo [1,2]†, Hiroko Okawa [1,2]†, Akishige Hokugo [1,3], Bhumika Shokeen [4], Oskar Sundberg [5], Yiying Zheng [5], Charles E. McKenna [5]*, Renate Lux [4]*, Ichiro Nishimura [1]*

1. Weintraub Center for Reconstructive Biotechnology, UCLA School of Dentistry, Los Angeles, CA 90095, USA
2. Division of Molecular and Regenerative Prosthodontics, Tohoku University Graduate School of Dentistry, Sendai, Miyagi 980-8575, Japan
3. Regenerative Bioengineering and Repair Laboratory, Division of Plastic and Reconstructive Surgery, Department of Surgery, David Geffen School of Medicine at UCLA, Los Angeles, CA 90095, USA
4. Section of Biosystems and Function, UCLA School of Dentistry, Los Angeles, CA 90095, USA
5. Department of Chemistry, Dana and David Dornsife College of Letters, Arts and Sciences, University of Southern California, Los Angeles, CA 90089, USA

† These co-authors contributed equally

*Co-corresponding Authors

Contact corresponding author:

Ichiro Nishimura, DDS, DMSc, DMD

Weintraub Center for Reconstructive Biotechnology

UCLA School of Dentistry

Box 951668, CHS B3-087, Los Angeles, CA 90095, USA

Tel: +1 (310) 794-7612, Fax: +1 (310) 825-6345

E-mail: inishimura@dentistry. ucla.edu

**Supplementary Fig. 1. Ligature-induced periodontitis in mice**

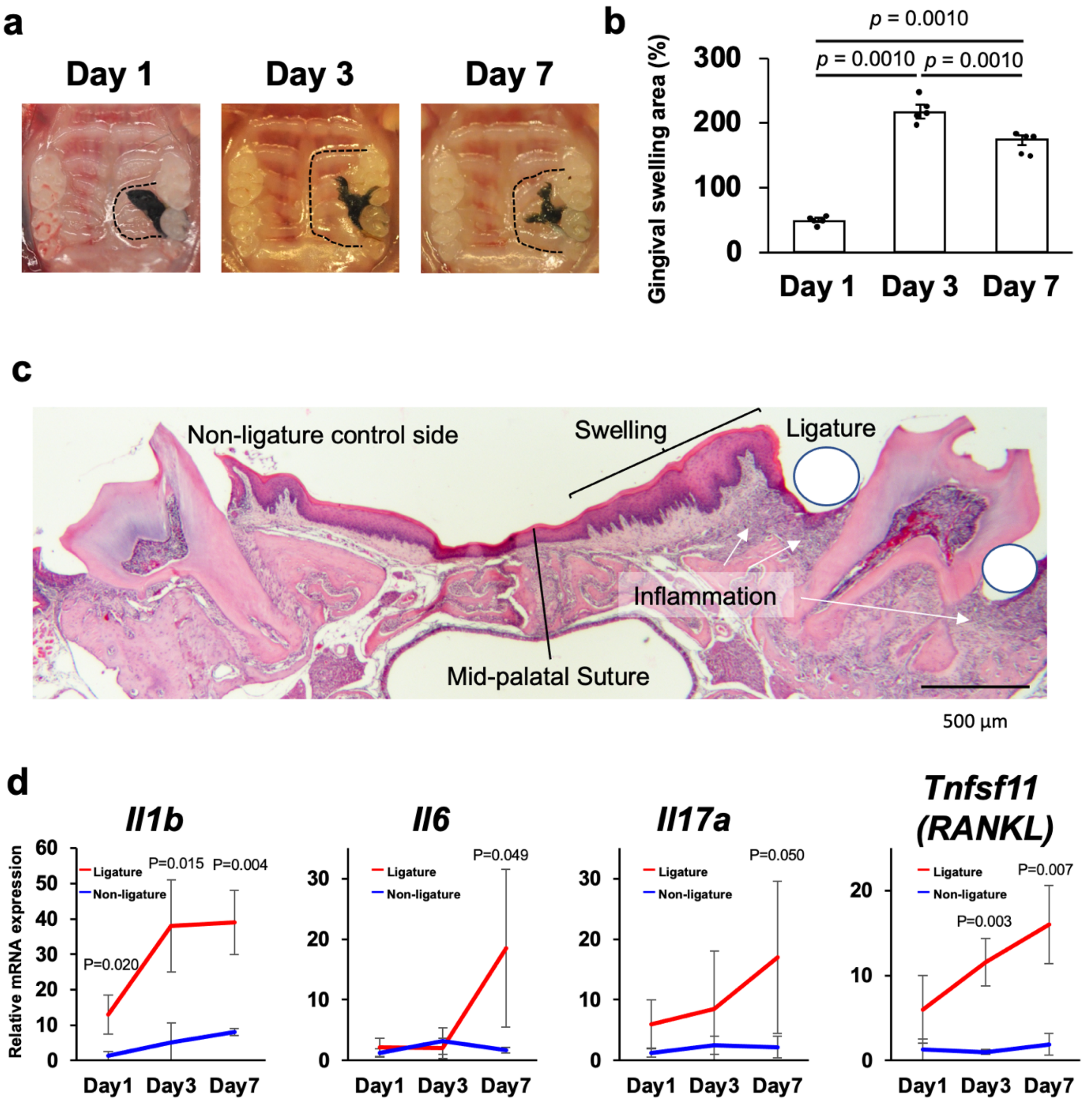


**a**. Representative images of the palate at 1, 3 and 7 days after ligature placement around the maxillary left second molar. The dotted lines demarcate gingival swelling. **b**. The gingival swelling area was measured as standardized by the circumferential area of the second molar (n = 4-5). **c**. Histological cross-section of maxilla depicting the second molar with ligature-induced gingival inflammation and swelling. The contralateral untreated second molar was associated with normal gingiva. **d**. Gene expression of *Il1b*, *Il6*, *Il17a* and *Tnfs11 (RANKL)* on the ligature side of the palatal gingival tissue was determined by quantitative real-time RT-PCR analysis. Gene expression of glyceraldehyde-3-phosphate dehydrogenase (*Gapdh*) was used as an internal control (n = 3). ANOVA with Tukey's multiple-comparison test (**b**) or Student's *t*-test (**c**) was used. Data are presented as mean values ± SD; $p < 0.05$ was considered significant. The source data are found in Supplementary Data S1, Fig. S1b and Fig. S1d.

## Supplementary Fig. 2. Chemical synthesis and characterization of OFS probes

**a**

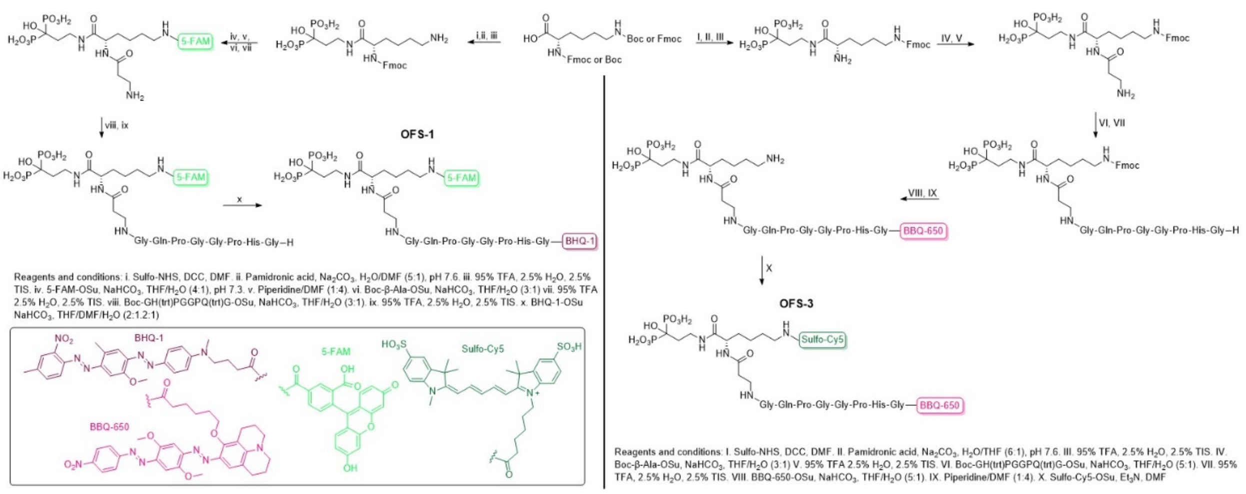


**b**

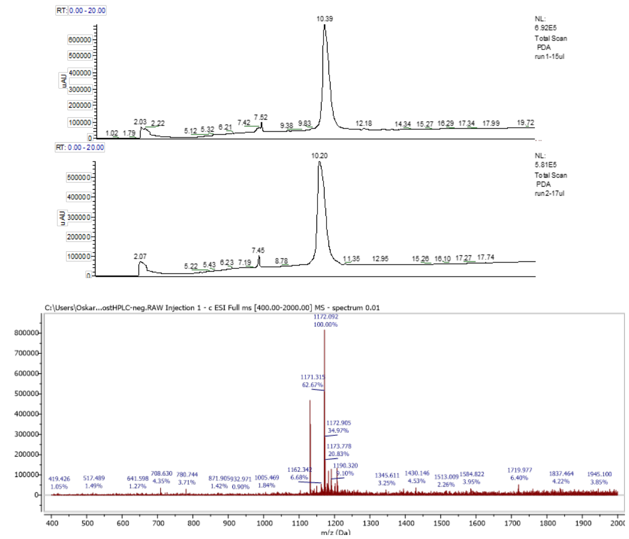


**c**

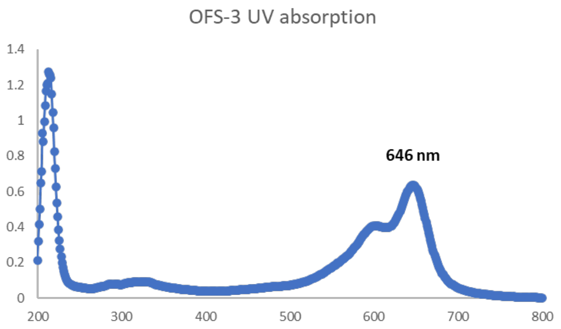


**a**. Chemical synthesis scheme of OFS-1 with 5-FAM/BHQ-1 and OFS-3 with Sulfo-Cy5/BBQ-650. (Adapted from Richard et al.[1])
**b**. LC-MS (Finnigan LCQ Deca XP Max equipped with an electrospray ionization (ESI) source and a photodiode array (PDA) detector) purity duplicate analysis of OFS-3 following HPLC purification (Shodex Asahipak ODP-50 4D column, 4.6 x 150 mm, 5 µm) using 50.0 mM TEAB buffers (A: 5% acetonitrile, pH 8.0, B: 75% acetonitrile, pH 8.0). Gradient: 0-1 min, 0% B; 1-20 min, 0-100% B; flow rate, 0.8 mL/min. OFS-3 had a retention time of 10.3 +/- 0.1 min. MS calcd for $C_{105}H_{138}N_{23}O_{31}P_2S_2$ $[M-2H]^{2-}$: 1171.9 m/z, found 1172.1 m/z. **c**. UV-Vis data of OFS-3 obtained using a Beckman Coulter DU 800 spectrophotometer. Observed $\lambda_{max}$: 646 nm, consistent with reported value for Sulfo-Cy5 (Lumiprobe).

**Supplementary Fig. 3. Depiction of mouse gingival cells by scRNA-seq**

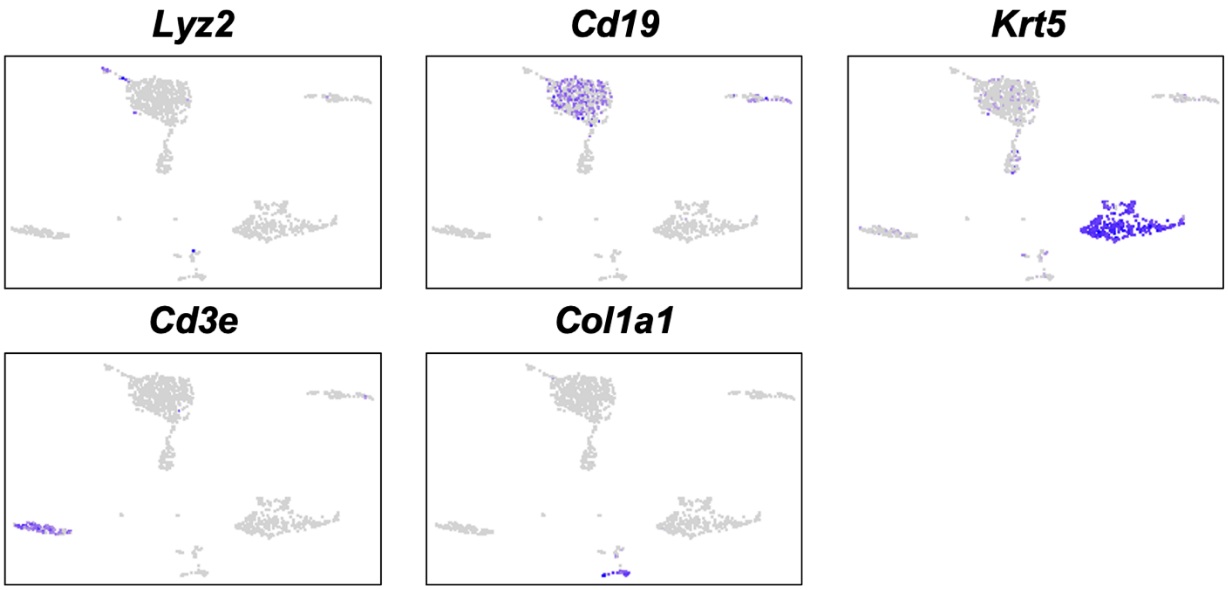


Single cell RNA sequencing (scRNA-seq) *t*-SNE projection plots showing transcript accumulation for *Lyz2*, *Cd19*, *Krt5*, *Cd3e* and *Collagen type I alpha 1 (Col1a1)* genes in individual cells clustering as myeloid cells, B cells, epithelial cells, T cells and fibroblasts, respectively. Color intensity indicates the relative gene expression level for the indicated gene in each cell (*Blue*: high expression, *gray*: no expression).

**Supplementary Fig. 4. Langerhans cells in scRNA-seq of control mouse gingiva**

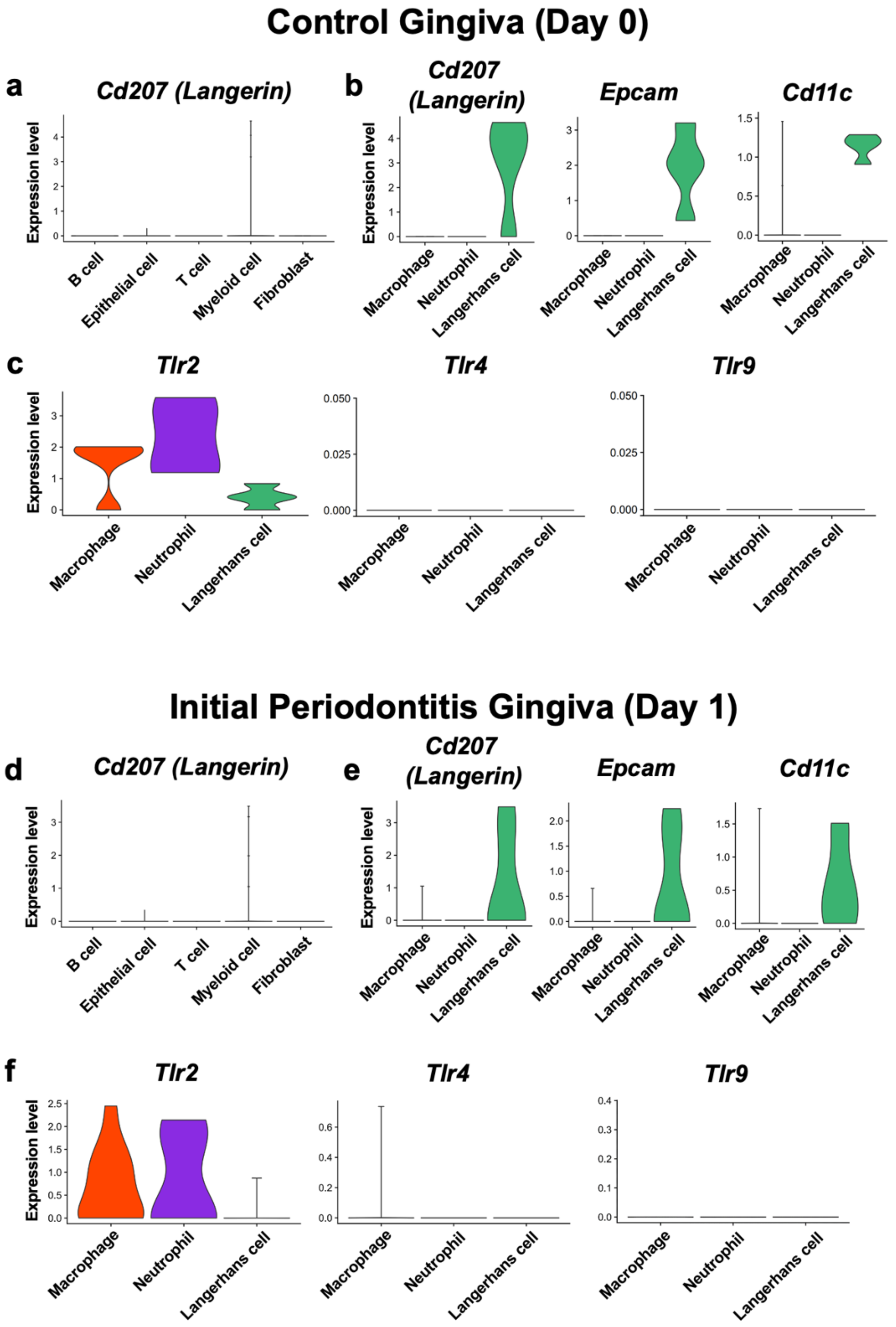


**a, d**. *Cd207* (*Langerin)* expressing Langerhans cells were suggested in the myeloid cell cluster.
**b, e**. The myeloid cell cluster was subclustered. One subcluster was associated with the selective expression of *Langerin*, *Epcam* and *Cd11c* and thus designated as Langerhans cells.
**c, f**. Myeloid cell subclusters exhibited the expression of *Tlr2* at different degrees. However, *Tlr4* and *Tlr9* expression was not detected by scRNA-seq.
**a, b, c** representing untreated control palatal gingiva: **d, e, f** representing initial periodontitis lesion of 1 day after the ligature placement.
