# Supplementary material for: Oral microbial extracellular DNA initiates periodontitis through gingival degradation by fibroblast-derived cathepsin K in mice
Source: Commun Biol. 2022 Sep 14;5:962. doi: 10.1038/s42003-022-03896-7 (PMC9474870; doi:10.1038/s42003-022-03896-7)
Supplement: Supplementary file 2 — Description of Additional Supplementary Data [file 42003_2022_3896_MOESM2_ESM.pdf]

Description of Additional Supplementary Files

File name: Supplementary Data 1
Description: Dataset of Fig. 1b, 1d, 1e and 1i

File name: Supplementary Data 2
Description: Dataset of Fig. 2b and 2d

File name: Supplementary Data 3
Description: Dataset of Fig. 3b and 3d

File name: Supplementary Data 4
Description: Dataset of Fig. 4b, 4c and 4d

File name: Supplementary Data 5
Description: Dataset of Fig. 5b

File name: Supplementary Data S1
Description: Dataset of Fig. S1b and S1d
