# Supplementary material for: Oral microbial extracellular DNA initiates periodontitis through gingival degradation by fibroblast-derived cathepsin K in mice
Source: Commun Biol. 2022 Sep 14;5:962. doi: 10.1038/s42003-022-03896-7 (PMC9474870; doi:10.1038/s42003-022-03896-7)
Supplement: Supplementary file 9 — Reporting Summary [file 42003_2022_3896_MOESM9_ESM.pdf]

nature research

Corresponding author(s): Ichiro Nishimura, Renate Lux, Charles E. McKenna

Last updated by author(s): Aug 9, 2022

Reporting Summary

Nature Research wishes to improve the reproducibility of the work that we publish. This form provides structure for consistency and transparency in reporting. For further information on Nature Research policies, see our Editorial Policies and the Editorial Policy Checklist.

Statistics

For all statistical analyses, confirm that the following items are present in the figure legend, table legend, main text, or Methods section.

n/a	Confirmed	
☐	☒	The exact sample size (*n*) for each experimental group/condition, given as a discrete number and unit of measurement
☐	☒	A statement on whether measurements were taken from distinct samples or whether the same sample was measured repeatedly
☐	☒	The statistical test(s) used AND whether they are one- or two-sided<br>*Only common tests should be described solely by name; describe more complex techniques in the Methods section.*
☒	☐	A description of all covariates tested
☒	☐	A description of any assumptions or corrections, such as tests of normality and adjustment for multiple comparisons
☐	☒	A full description of the statistical parameters including central tendency (e.g. means) or other basic estimates (e.g. regression coefficient) AND variation (e.g. standard deviation) or associated estimates of uncertainty (e.g. confidence intervals)
☒	☐	For null hypothesis testing, the test statistic (e.g. *F*, *t*, *r*) with confidence intervals, effect sizes, degrees of freedom and *P* value noted<br>*Give P values as exact values whenever suitable.*
☒	☐	For Bayesian analysis, information on the choice of priors and Markov chain Monte Carlo settings
☒	☐	For hierarchical and complex designs, identification of the appropriate level for tests and full reporting of outcomes
☒	☐	Estimates of effect sizes (e.g. Cohen's *d*, Pearson's *r*), indicating how they were calculated

Our web collection on statistics for biologists contains articles on many of the points above.

Software and code

Policy information about availability of computer code

Data collection	scRNA-seq was performed with a commercially available platform (10X Genomics, San Francisco, CA).
Data analysis	The Cell Ranger output of scRNA-seq data were analyzed using an R toolkit for single cell genomics (Seurat, https://satijalab.org/seurat/).

For manuscripts utilizing custom algorithms or software that are central to the research but not yet described in published literature, software must be made available to editors and reviewers. We strongly encourage code deposition in a community repository (e.g. GitHub). See the Nature Research guidelines for submitting code & software for further information.

Data

Policy information about availability of data

All manuscripts must include a data availability statement. This statement should provide the following information, where applicable:

- Accession codes, unique identifiers, or web links for publicly available datasets
- A list of figures that have associated raw data
- A description of any restrictions on data availability

We state where data supporting the results reported in a published article can be found. Single cell RNA sequence data are available at GEO (access code: GSE201108 and GSE201109).

Field-specific reporting

Please select the one below that is the best fit for your research. If you are not sure, read the appropriate sections before making your selection.

☒ Life sciences ☐ Behavioural & social sciences ☐ Ecological, evolutionary & environmental sciences

For a reference copy of the document with all sections, see nature.com/documents/nr-reporting-summary-flat.pdf

Life sciences study design

All studies must disclose on these points even when the disclosure is negative.

Sample size	No sample size calculation was performed. The sample size was selected from the published data that used the similar experimental protocols.
Data exclusions	We did not exclude data from our analyses.
Replication	Each experiment contained repeated controls ensuring the reproducibility.
Randomization	In animal study, animals were randomly assigned to the experimental groups.
Blinding	N/a

Reporting for specific materials, systems and methods

We require information from authors about some types of materials, experimental systems and methods used in many studies. Here, indicate whether each material, system or method listed is relevant to your study. If you are not sure if a list item applies to your research, read the appropriate section before selecting a response.

Materials & experimental systems

n/a	Involved in the study	
☐	☒	Antibodies
☒	☐	Eukaryotic cell lines
☒	☐	Palaeontology and archaeology
☐	☒	Animals and other organisms
☐	☒	Human research participants
☒	☐	Clinical data
☒	☐	Dual use research of concern

Methods

n/a	Involved in the study	
☒	☐	ChIP-seq
☒	☐	Flow cytometry
☒	☐	MRI-based neuroimaging

Antibodies

Antibodies used	Immunohistochemistry. Anti-Ctsk antibody (1:100) and Anti-col14a1 antibody (1:1000).
Validation	We used commercially available antibodies and we relied on commercial validation.

Animals and other organisms

Policy information about studies involving animals; ARRIVE guidelines recommended for reporting animal research

Laboratory animals	Mice, C57Bl6, Female, 8-10 weeks old
Wild animals	N/a
Field-collected samples	N/a
Ethics oversight	All protocols for animal experiments were reviewed and approved by the University of California Los Angeles (UCLA) Animal Research Committee (ARC# 2003-009) and followed the Public Health Service Policy for the Humane Care and Use of Laboratory Animals and the UCLA Animal Care and Use Training Manual guidelines.

Note that full information on the approval of the study protocol must also be provided in the manuscript.

Human research participants

Policy information about studies involving human research participants

Population characteristics	Saliva samples were collected from 20 to 40-year old healthy human subjects. Sub-gingival plaque samples were collected from 20 to 40-year old human subjects with clinical diagnosis of periodontitis.
Recruitment	After verbal consent was obtained, human participants were recruited in the UCLA School of Dentistry clinic.
Ethics oversight	All protocols involving human subjects were reviewed and approved by the UCLA Institutional Review Board (UCLA-IRB 11-002483).

Note that full information on the approval of the study protocol must also be provided in the manuscript.
